# Genome Sequence of *Novoherbaspirillum* sp. UKPF54, a Plant Growth-Promoting Rhizobacterial Strain with N_2_O-Mitigating Abilities, Isolated from Paddy Soil

**DOI:** 10.1128/MRA.00999-19

**Published:** 2020-01-16

**Authors:** Nan Gao, Chaowei Zhou, Weishou Shen, Sayuri Ota, Yutaka Shiratori, Tomoyasu Nishizawa, Kazuo Isobe, Xinhua He, Hanjie Ying, Keishi Senoo

**Affiliations:** a National Engineering Research Center for Biotechnology and School of Biological and Pharmaceutical Engineering, Nanjing Tech University, Nanjing, China; b Jiangsu Key Laboratory of Atmospheric Environment Monitoring and Pollution Control, Collaborative Innovation Center of Atmospheric Environment and Equipment Technology, and School of Environmental Science and Engineering, Nanjing University of Information Science and Technology, Nanjing, China; c Niigata Agricultural Research Institute, Niigata, Japan; d Department of Bioresource Science, College of Agriculture, Ibaraki University, Ibaraki, Japan; e Department of Applied Biological Chemistry, Graduate School of Agricultural and Life Sciences, The University of Tokyo, Tokyo, Japan; f Centre of Excellence for Soil Biology, College of Resources and Environment, Southwest University, Chongqing, China; g Collaborative Research Institute for Innovative Microbiology, The University of Tokyo, Tokyo, Japan; University of California, Riverside

## Abstract

*Novoherbaspirillum* sp. strain UKPF54, a plant growth-promoting rhizobacterium with the ability to mitigate nitrous oxide emission from agriculture soils, has been successfully isolated from paddy soil in Kumamoto, Japan. Here, we report the whole-genome sequence of this strain.

## ANNOUNCEMENT

*Herbaspirillum* spp. are endophytic diazotrophs which can colonize sugarcane, rice, maize, sorghum, red clover, and other crops (1–3). Some *Herbaspirillum* sp. strains and their close relatives, such as *Novoherbaspirillum* sp. strains, are known to promote plant growth, suggesting that they are important resource species for the development of biofertilizers (1–3). *Novoherbaspirillum* sp. strain UKPF54, previously called *Herbaspirillum* sp. strain UKPF54, was isolated from the rhizosphere of paddy soil in Kumamoto, Japan (4). It simultaneously promotes the growth of pasture plants and mitigates nitrous oxide emissions from soils (3, 5).

*Novoherbaspirillum* sp. UKPF54 was grown in 5 ml NBNS culture medium (briefly, 5 g liter^−1^ peptone and 3 g liter^−1^ beef extract containing 0.3 mM NaNO_3_ and 4 mM sodium succinate [pH 7.0]) at 28°C and 220 rpm. The genomic DNA was extracted using a DNeasy blood and tissue kit (Qiagen, Germany). A SMRTbell library with a 20-kb insert size was constructed with the template prep kit 1.0 and the BluePippin size selection system, according to the manual. The genome was sequenced with the PacBio RS II DNA sequencing system using SMRT Cell 8Pac v3 and DNA polymerase binding kit P6 reagents. To remove the PacBio short reads, the RS HGAP assembly software (v3.0) was applied, with default parameters (6). When 5′ and 3′ ends were connected, the contig was assembled into a single circular DNA molecule. The circular DNA molecule had a mean subread length of 8,279 bp, an *N*_50_ value of raw sequences of 11,260 bp, and a total of 1,453,750,364 bases and 175,579 reads. *Novoherbaspirillum* sp. UKPF54 contains a chromosome of 4,718,988 bp with a G+C content of 61.89%. The sequencing depth reached 198×.

Genes were predicted using the NCBI Prokaryotic Genome Annotation Pipeline (PGAP, revision 4.8) with the best-placed reference protein set (GeneMarkS2+) (7, 8). In addition, BlastKOALA (9) against the “species_prokaryotes” database was used for functional annotation and KEGG pathway mapping. A total of 4,307 protein-coding sequences, 56 tRNAs, 9 rRNAs, 4 noncoding RNAs (ncRNAs), and 50 pseudogenes were discovered. The predicted functional genes consisted of 11 genes of the ABC transporters, 10 genes of the two-component system, and 5 genes of bacterial chemotaxis. The functional genes contained candidate genes associated with nitrogen metabolism and plant growth promotion (Table 1).

**TABLE 1:** Predicted genes associated with nitrogen metabolism and plant growth promotion in the *Novoherbaspirillum* sp. UKPF54 genome

Gene function	Gene name	Product^*a*^	Protein accession no.	Inference amino acid sequence ID^*b*^
Nitrogen fixation	*fdx*	ISC system 2Fe-2S type ferredoxin	QDZ27364	WP_012079114
	*fdxH*	Formate dehydrogenase subunit beta	QDZ27883	WP_018604908
	*fixA*	Electron transfer flavoprotein subunit beta/FixA family protein	QDZ26876	WP_015436355
	*fixB*	Electron transfer flavoprotein subunit alpha/FixB family protein	QDZ26877	WP_008445988
	*nifU*	SUF system NifU family Fe-S cluster assembly protein	QDZ29484	WP_018310943
Nitrate utilization	*napA*	Nitrate reductase catalytic subunit NapA	QDZ28521	YP_006896445
	*napE*	Nitrate reductase	QDZ28519	WP_006394233
	*narG*	Nitrate reductase subunit alpha	QDZ28003	WP_018151853
	*narH*	Nitrate reductase subunit beta	QDZ28002	WP_011871146
	*narI*	Respiratory nitrate reductase subunit gamma	QDZ28000	WP_011871144
	*narJ*	Nitrate reductase molybdenum cofactor assembly chaperone	QDZ28001	WP_008953006
Nitrite utilization	*nirB*	Nitrite reductase large subunit	QDZ29679	WP_017876120
	*nirD*	Nitrite reductase small subunit NirD	QDZ26547	WP_011829911
	*nirK*	Nitrite reductase, copper containing	QDZ28940	WP_013213801
Nitrous oxide reduction	*nosD*	Nitrous oxide reductase family maturation protein NosD	QDZ30537	WP_017879062
	*nosL*	Nitrous oxide reductase accessory protein NosL	QDZ30535	WP_018077130
	*nosZ*	Nitrous oxide reductase	QDZ28019	WP_019898632
Nitrogen regulation	*fnr*	Fumarate/nitrate reduction transcriptional regulator Fnr	QDZ27979	WP_011871123
	*ntrC*	Nitrogen regulation protein NR(I)	QDZ28461	WP_019141540
Indole acetic acid synthesis	*trpC*	Indole-3-glycerol phosphate synthase TrpC	QDZ30261	WP_005671473
Phosphate solubilization	*pqqB*	Pyrroloquinoline quinone biosynthesis protein	QDZ26968	WP_013691667
Acetolactate synthesis	*ilvB*	Acetolactate synthase 3 catalytic subunit	QDZ28257	WP_016832326
	*ilvC*	Ketol-acid reductoisomerase	QDZ28255	WP_003261913
	*ilvD*	Dihydroxy-acid dehydratase	QDZ28620	WP_007877043
	*ilvN*	Acetolactate synthase small subunit	QDZ28256	WP_004630835

a ISC, iron-sulfur cluster; SUF, sulfur assimilation.

b NCBI accession number of most similar protein sequence from which function is inferred. ID, identification.

We analyzed the secondary metabolism cluster of the complete genome with antiSMASH v5.0.0 (10). The results showed that four secondary metabolite gene clusters relevant to the production of active substances were predicted. Moreover, two nonribosomal peptide synthetase (NRPS)-like fragment gene clusters and one beta-lactone-containing protease inhibitor (betalactone) gene cluster were predicted.

Overall, the whole-genome sequence is of critical importance to reveal the molecular mechanisms for the promotion of plant growth and the mitigation of nitrous oxide from agricultural soils by *Novoherbaspirillum* sp. UKPF54, thereby providing fundamental support to develop biofertilizer applications with this strain.

### Data availability.

The whole-genome sequence of *Novoherbaspirillum* sp. UKPF54 has been deposited in GenBank under the accession number CP040128. The raw reads have been deposited in the Sequence Read Archive (SRA) under the accession number SRR8943564.
